# There and back again: insulin, ENaC, and the cortical collecting duct

**DOI:** 10.14814/phy2.12809

**Published:** 2016-05-27

**Authors:** Alan C. Pao

**Affiliations:** ^1^ Department of MedicineStanford UniversityStanfordCalifornia

## Abstract

Cell culture models suggest mechanisms by which insulin stimulates ENaC in the cortical collecting duct. These mechanisms still need to be tested for physiological significance in animal models of insulin resistance.

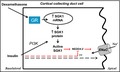

“There is nothing like looking, if you want to find something. You certainly usually find something, if you look, but it is not always quite the something you were after.”

– *The Hobbit, or There and Back Again* by J.R.R. Tolkien

The epithelial sodium channel (ENaC) is a heteromeric channel expressed in the principal cell of the cortical collecting duct and provides the final pathway for Na^+^ reabsorption before tubular fluid becomes urine. Because ENaC is in a strategic position to dictate the Na^+^ composition of tubular fluid, it is subject to extensive regulation by numerous factors including mechanical forces, proteases, ions, and hormones (Bhalla and Hallows 2008). Cell culture models of the cortical collecting duct have provided a wealth of information about how steroid hormones, such as aldosterone, and growth factors, such as insulin and IGF‐1, control ENaC activity (Chen et al. 1998, 1999; Record et al. 1998; Naray‐Fejes‐Toth et al. 1999; Wang et al. 2001; Gonzalez‐Rodriguez et al. 2007; Pavlov et al. 2013). However, some cell culture models do not always recapitulate cortical collecting duct function perfectly. For example, X*enopus laevis* A6 cells and murine M1 cells exhibit morphological and electrical properties of the cortical collecting duct, yet both cell lines lack transcriptionally competent mineralocorticoid receptors (Chen et al. 1998). This is significant because mineralocorticoid receptors respond to aldosterone, a key hormone that regulates ENaC activity, extracellular fluid volume, and blood pressure. Supraphysiological doses of aldosterone (in the μmol/L range) are often required to stimulate ENaC in cultured collecting duct cells, and glucocorticoids, such as dexamethasone, are sometimes used to probe mineralocorticoid function because glucocorticoids activate signal transduction cascades that are shared by both glucocorticoid and mineralocorticoid receptors. Supraphysiological doses of insulin (in the 30–50 nmol/L range) are also occasionally needed to stimulate ENaC in cultured collecting duct cells, where IGF type 1 receptors instead of insulin receptors are likely activated (Gonzalez‐Rodriguez et al. 2007).

In this issue of *Physiological Reports*, Mansley et al. (2016) revisit the mechanisms by which dexamethasone and insulin stimulate ENaC in murine mpkCCD_c14_ cells. The investigators demonstrate that dexamethasone (100 nmol/L) and insulin (20 nmol/L) both rapidly (within 45 min of administration) stimulate ENaC, but these hormones do so through distinct mechanisms (Fig. 1). Dexamethasone increases SGK1 (serum‐ and glucocorticoid‐regulated kinase 1) mRNA and protein expression, but dexamethasone does not stimulate cellular PI3K (phosphatidylinositol 3‐kinase) activity as detected by the expression of phosphorylated forms of PKB (protein kinase B). The investigators suggest that the level of SGK1 expression is the rate‐limiting step for dexamethasone‐mediated stimulation of ENaC and that the basal level of PI3K activity is sufficient to phosphorylate and activate SGK1. Together, these findings confirm prior studies establishing SGK1 as a prototypic early‐response gene and key mediator of steroid hormone action on ENaC in cortical collecting duct cells (Chen et al. 1999; Naray‐Fejes‐Toth et al. 1999).

**Figure 1:**
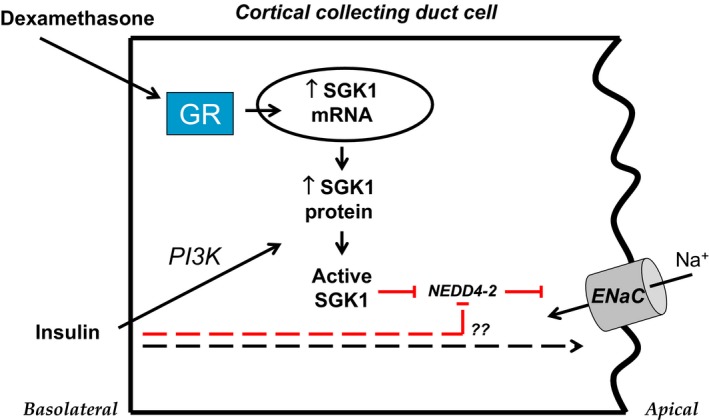
Model of insulin action in the cortical collecting duct. In cultured cortical collecting duct cells, dexamethasone activates glucocorticoid receptors (GR) which rapidly stimulate transcription of SGK1 mRNA and translation of SGK1 protein. Insulin or IGF‐1 (not pictured) activates PI3K which induces phosphorylation and activation of SGK1 protein. Once activated, SGK1 phosphorylates and inhibits the E3 ubiquitin ligase Nedd4‐2. Under basal conditions, Nedd4‐2 decreases expression of ENaC in the apical cell membrane by promoting channel ubiquitination and retrieval. Upon inhibition of Nedd4‐2 by SGK1, ENaC residency in the apical membrane increases, leading to apical entry of Na^+^ into the cell. A parallel pathway may enable insulin to stimulate ENaC in the absence of PI3K or SGK1 activity (dashed lines). Stimulatory pathways are delineated in black; inhibitory pathways are in red.

Mansley et al. further demonstrate that insulin stimulates ENaC, but it does so by stimulating cellular PI3K and SGK1 activity instead of SGK1 expression (Mansley et al. 2016). Interestingly, when mpkCCD_c14_ cells are pretreated with cyclohexamide, insulin‐stimulated cellular PI3K or SGK1 activity is eliminated, yet insulin‐stimulated ENaC current remains unaffected. These findings are surprising because it contradicts prior work showing the following: (1) inhibiting PI3K blocks insulin or IGF‐1‐stimulated ENaC current (Record et al. 1998; Gonzalez‐Rodriguez et al. 2007; Pavlov et al. 2013); and (2) inhibiting PI3K abolishes insulin or IGF‐1‐induced phosphorylation and activation of SGK1 (Wang et al. 2001; Gonzalez‐Rodriguez et al. 2007). It is notable that Mansfield et al. have not yet confirmed whether pretreatment of cells with a direct PI3K inhibitor would yield similar results, nor have they evaluated how insulin might stimulate ENaC in the absence of PI3K activity. It is possible that cyclohexamide, a protein synthesis inhibitor, inhibits an array of signaling pathways that ultimately preserves insulin‐stimulated ENaC current regardless of the status of PI3K activity. Nonetheless, there is evidence for dissociation between insulin‐stimulated PI3K activation and insulin‐stimulated PKB activity in insulin‐resistant adipocytes and skeletal muscle (Kim et al. 1999a,b; Nadler et al. 2001; Kruszynska et al. 2002), leaving open the possibility that there may be genuine dissociation between insulin‐stimulated PI3K/SGK1 activation and insulin‐stimulated ENaC current.

While the notion that insulin can stimulate ENaC via SGK1 is not new, the physiological significance of such signaling is still not clear. Global deletion of SGK1 in mice prevents high blood pressure that is induced by high fat feeding, a commonly used maneuver to induce insulin resistance (Huang et al. 2006); however, this study did not explicitly examine whether elevated ENaC activity was responsible for high‐fat diet‐induced high blood pressure (Huang et al. 2006). Indeed, the question of whether ENaC mediates renal Na^+^ retention and high blood pressure in the context of insulin resistance is still controversial. Acute administration of insulin to rats induces apical localization of ENaC in the cortical collecting duct (Tiwari et al. 2007). Moreover, deletion of insulin receptors from the collecting duct in mice decreases ENaC activity (Pavlov et al. 2013), implying that hyperinsulinemia would lead to elevated ENaC activity. However, acute administration of insulin, at concentrations found in insulin‐resistant humans and rodents, fails to increase ENaC activity in split‐open rat cortical collecting duct (Frindt and Palmer 2012). Finally, Nizar et al. have assiduously demonstrated that ENaC does not mediate renal Na^+^ retention or salt‐sensitive increases in blood pressure in a high fat‐fed mouse model of insulin resistance (Nizar et al. 2016). Collectively, these in vivo studies underscore the importance of testing the physiological significance of insulin‐ENaC signaling in animal models of insulin resistance.

Returning to this study, Mansley et al. may have identified a new path by which insulin stimulates ENaC that is independent of PI3K. If verified with experiments that directly inhibit PI3K, this finding raises new questions about how insulin might stimulate ENaC in the absence of PI3K activity. What are the parallel pathways that enable the dissociation between insulin‐stimulated PI3K/SGK1 activation and insulin‐stimulated ENaC current? Does the level of PI3K activity change in the cortical collecting duct under conditions of insulin resistance? If insulin can stimulate ENaC independent of PI3K, does a decrease in PI3K activity in the cortical collecting duct lead to a decrease renal Na^+^ retention or blood pressure? Such questions are best answered back again in animal models of insulin resistance, where these pathways can be characterized under the appropriate pathophysiological contexts and their physiological significance can be properly ascertained.

## References

[phy212809-bib-0001] Bhalla, V. , and K. R. Hallows . 2008 Mechanisms of ENaC regulation and clinical implications. J. Am. Soc. Nephrol. 19:1845–1854. 18753254 10.1681/ASN.2008020225

[phy212809-bib-0002] Chen, S. Y. , J. Wang , W. Liu , and D. Pearce . 1998 Aldosterone responsiveness of A6 cells is restored by cloned rat mineralocorticoid receptor. Am. J. Physiol. 274:C39–C46. 9458711 10.1152/ajpcell.1998.274.1.C39

[phy212809-bib-0003] Chen, S. Y. , A. Bhargava , L. Mastroberardino , O. C. Meijer , J. Wang , P. Buse , et al. 1999 Epithelial sodium channel regulated by aldosterone‐induced protein sgk. Proc. Natl Acad. Sci. U. S. A. 96:2514–2519. 10051674 10.1073/pnas.96.5.2514 PMC26816

[phy212809-bib-0004] Frindt, G. , and L. G. Palmer . 2012 Effects of insulin on Na and K transporters in the rat CCD. Am. J. Physiol. Renal. Physiol. 302:F1227–F1233. 22357918 10.1152/ajprenal.00675.2011 PMC3362065

[phy212809-bib-0005] Gonzalez‐Rodriguez, E. , H. P. Gaeggeler , and B. C. Rossier . 2007 IGF‐1 vs insulin: respective roles in modulating sodium transport via the PI‐3 kinase/Sgk1 pathway in a cortical collecting duct cell line. Kidney Int. 71:116–125. 17164836 10.1038/sj.ki.5002018

[phy212809-bib-0006] Huang, D. Y. , K. M. Boini , H. Osswald , B. Friedrich , F. Artunc , S. Ullrich , et al. 2006 Resistance of mice lacking the serum‐ and glucocorticoid‐inducible kinase SGK1 against salt‐sensitive hypertension induced by a high‐fat diet. Am. J. Physiol. Renal. Physiol. 291:F1264–F1273. 17003223 10.1152/ajprenal.00299.2005

[phy212809-bib-0007] Kim, Y. B. , S. E. Nikoulina , T. P. Ciaraldi , R. R. Henry , and B. B. Kahn . 1999a Normal insulin‐dependent activation of Akt/protein kinase B, with diminished activation of phosphoinositide 3‐kinase, in muscle in type 2 diabetes. J. Clin. Investig. 104:733–741. 10491408 10.1172/JCI6928 PMC408433

[phy212809-bib-0008] Kim, Y. B. , J. S. Zhu , J. R. Zierath , H. Q. Shen , A. D. Baron , and B. B. Kahn . 1999b Glucosamine infusion in rats rapidly impairs insulin stimulation of phosphoinositide 3‐kinase but does not alter activation of Akt/protein kinase B in skeletal muscle. Diabetes 48:310–320. 10334307 10.2337/diabetes.48.2.310

[phy212809-bib-0009] Kruszynska, Y. T. , D. S. Worrall , J. Ofrecio , J. P. Frias , G. Macaraeg , and J. M. Olefsky . 2002 Fatty acid‐induced insulin resistance: decreased muscle PI3K activation but unchanged Akt phosphorylation. J. Clin. Endocrinol. Metab. 87:226–234. 11788651 10.1210/jcem.87.1.8187

[phy212809-bib-0111] Mansley, M. K. , G. B. Watt , S. L. Francis , D. J. Walker , S. C. Land , M. A. Bailey , et al. 2016 Dexamethasone and insulin activate serum and glucocorticoid‐inducible kinase 1 (SGK1) via different molecular mechanisms in cortical collecting duct cells. Physiol. Rep. doi: 10.14814/phy2.12792. 10.14814/phy2.12792 PMC4886164 27225626

[phy212809-bib-0010] Nadler, S. T. , J. P. Stoehr , M. E. Rabaglia , K. L. Schueler , M. J. Birnbaum , and A. D. Attie . 2001 Normal Akt/PKB with reduced PI3K activation in insulin‐resistant mice. Am. J. Physiol. Endocrinol. Metab. 281:E1249–E1254. 11701440 10.1152/ajpendo.2001.281.6.E1249

[phy212809-bib-0011] Naray‐Fejes‐Toth, A. , C. Canessa , E. S. Cleaveland , G. Aldrich , and G. Fejes‐Toth . 1999 SGK is an aldosterone‐induced kinase in the renal collecting duct. Effects on epithelial Na+ channels. J. Biol. Chem. 274:16973–16978. 10358046 10.1074/jbc.274.24.16973

[phy212809-bib-0012] Nizar, J. M. , W. Dong , R. B. McClellan , M. Labarca , Y. Zhou , J. Wong , et al. 2016 Sodium‐sensitive elevation in blood pressure is ENaC independent in diet‐induced obesity and insulin resistance. Am. J. Physiol. Renal. Physiol. 00265:02015. 10.1152/ajprenal.00265.2015 PMC4867314 26841823

[phy212809-bib-0013] Pavlov, T. S. , D. V. Ilatovskaya , V. Levchenko , L. Li , C. M. Ecelbarger , and A. Staruschenko . 2013 Regulation of ENaC in mice lacking renal insulin receptors in the collecting duct. FASEB J. 27:2723–2732. 23558339 10.1096/fj.12-223792 PMC3688749

[phy212809-bib-0014] Record, R. D. , L. L. Froelich , C. J. Vlahos , and B. L. Blazer‐Yost . 1998 Phosphatidylinositol 3‐kinase activation is required for insulin‐stimulated sodium transport in A6 cells. Am. J. Physiol. 274:E611–E617. 9575821 10.1152/ajpendo.1998.274.4.E611

[phy212809-bib-0015] Tiwari, S. , L. Nordquist , V. K. Halagappa , and C. A. Ecelbarger . 2007 Trafficking of ENaC subunits in response to acute insulin in mouse kidney. Am. J. Physiol. Renal. Physiol. 293:F178–F185. 17389677 10.1152/ajprenal.00447.2006

[phy212809-bib-0016] Wang, J. , P. Barbry , A. C. Maiyar , D. J. Rozansky , A. Bhargava , M. Leong , et al. 2001 SGK integrates insulin and mineralocorticoid regulation of epithelial sodium transport. Am. J. Physiol. Renal. Physiol. 280:F303–F313. 11208606 10.1152/ajprenal.2001.280.2.F303

